# Editorial Note: Methionine-induced regulation of growth, secondary metabolites and oxidative defense system in sunflower (*Helianthus annuus *L.) plants subjected to water deficit stress

**DOI:** 10.1371/journal.pone.0318868

**Published:** 2025-01-30

**Authors:** The PLOS One Editors

Following the publication and subsequent retraction of this article [1, 2], PLOS was informed by the Universitat Politècnica de València that co-author PK left the institute in May 2019. Corresponding author PA commented that the study was conducted between 2020–2021, indicating that the affiliation listed for co-author PK is incorrect.

This Editorial Note is issued to inform readers that the correct affiliation for co-author PK should read “Independent Researcher, Valencia, Spain” instead.
